# Small-molecule HDAC and Akt inhibitors suppress tumor growth and enhance immunotherapy in multiple myeloma

**DOI:** 10.1186/s13046-021-01909-7

**Published:** 2021-03-23

**Authors:** Mitsuhito Hirano, Yoichi Imai, Yuta Kaito, Takahiko Murayama, Kota Sato, Tadao Ishida, Junichi Yamamoto, Takumi Ito, Muneyoshi Futami, Masaki Ri, Hiroshi Yasui, Tamami Denda, Yukihisa Tanaka, Yasunori Ota, Masanori Nojima, Yasuhiko Kamikubo, Noriko Gotoh, Shinsuke Iida, Hiroshi Handa, Arinobu Tojo

**Affiliations:** 1 Division of Molecular Therapy, Advanced Clinical Research Center, The Institute of Medical Science, The University of Tokyo, Tokyo, Japan; 2 Department of Hematology/Oncology, Research Hospital, The Institute of Medical Science, The University of Tokyo, Tokyo, Japan; 3 Division of Cancer Cell Biology, Cancer Research Institute of Kanazawa University, Kanazawa, Japan; 4 Department of Hematology, Japanese Red Cross Medical Center, Tokyo, Japan; 5 School of Life Science and Technology, Tokyo Institute of Technology, Tokyo, Japan; 6 Department of Chemical Biology, Tokyo Medical University, Tokyo, Japan; 7 Department of Hematology & Oncology, Nagoya City University Graduate School of Medical Sciences, Nagoya, Japan; 8 Project Division of Fundamental Study on Cutting Edge of Genome Medicine, The Institute of Medical Science, The University of Tokyo, Tokyo, Japan; 9 Department of Pathology, Research Hospital, The Institute of Medical Science, The University of Tokyo, Tokyo, Japan; 10 Center for Translational Research/Division of Advanced Medicine Promotion The Institute of Medical Science, The University of Tokyo, Tokyo, Japan; 11 Laboratory of Oncology and Strategic Innovation, Laboratory Science, Graduate School of Medicine Kyoto University, Kyoto, Japan

**Keywords:** Multiple myeloma, Cereblon, Drug-resistance, HDAC inhibitor, Akt inhibitor, Dual HDAC and PI3K inhibitor, Natural killer group 2D ligands, C-Myc, Monoclonal antibody, Antibody-dependent cellular cytotoxicity

## Abstract

**Background:**

Multiple myeloma (MM) is an incurable disease. The acquisition of resistance to drugs, including immunomodulatory drugs (IMiDs), has a negative effect on its prognosis. Cereblon (CRBN) is a key mediator of the bioactivities of IMiDs such as lenalidomide. Moreover, genetic alteration of CRBN is frequently detected in IMiD-resistant patients and is considered to contribute to IMiD resistance. Thus, overcoming resistance to drugs, including IMiDs, is expected to improve clinical outcomes. Here, we examined potential mechanisms of a histone deacetylase (HDAC) inhibitor and Akt inhibitor in relapsed/refractory MM patients.

**Methods:**

We established lenalidomide-resistant cells by knocking down CRBN with RNAi-mediated downregulation or knocking out CRBN using CRISPR-Cas9 in MM cells. Additionally, we derived multi-drug (bortezomib, doxorubicin, or dexamethasone)-resistant cell lines and primary cells from relapsed/refractory MM patients. The effects of HDAC and Akt inhibitors on these drug-resistant MM cells were then observed with a particular focus on whether HDAC inhibitors enhance immunotherapy efficacy. We also investigated the effect of lenalidomide on CRBN-deficient cells.

**Results:**

The HDAC inhibitor suppressed the growth of drug-resistant MM cell lines and enhanced the antibody-dependent cellular cytotoxicity (ADCC) of therapeutic antibodies by upregulating natural killer group 2D (NKG2D) ligands in MM cells. CRBN-deficient cells showed lenalidomide-induced upregulation of phosphorylated glycogen synthase kinase-3 (p-GSK-3) and c-Myc phosphorylation. Moreover, HDAC and Akt inhibitors downregulated c-Myc by blocking GSK-3 phosphorylation. HDAC and Akt inhibitors also exhibited synergistic cytotoxic and c-Myc-suppressive effects. The dual HDAC and PI3K inhibitor, CUDC-907, exhibited cytotoxic and immunotherapy-enhancing effects in MM cells, including multi-drug-resistant lines and primary cells from lenalidomide-resistant patients.

**Conclusions:**

The combination of an HDAC and an Akt inhibitor represents a promising approach for the treatment of relapsed/refractory MM.

**Supplementary Information:**

The online version contains supplementary material available at 10.1186/s13046-021-01909-7.

## Background

Multiple myeloma (MM) is a plasma cell malignancy that accounts for approximately 10% of all hematological malignancies [1–3]. Currently, there is no curative therapy for this disease, which is accompanied by symptoms including renal failure, anemia, hypercalcemia, and skeletal destruction [2]. However, the response rate in patients with MM has been improved by the introduction of various novel drugs available for MM treatment, including monoclonal antibodies (mAbs) such as daratumumab [4] and elotuzumab [5]. In addition, proteasome inhibitors (PIs), such as bortezomib [6] and immunomodulatory drugs (IMiDs), including thalidomide [3], lenalidomide [7], and pomalidomide [8], have shown efficacy in MM patients. Notably, the combined use of lenalidomide with PIs [9] and/or mAbs [10] is a key approach in current MM therapy [11].

Most patients, including those that responded to these drugs, developed drug resistance; therefore, it is essential to overcome this issue to improve survival. Among the novel drugs described, IMiDs are supposed to be key drugs as partners of PIs and mAbs for use in combination therapy [2]. Cereblon (CRBN) is a component of the CUL4 E3 ligase complex that serves as the primary target of IMiDs [12–14]. Reduced CRBN expression is thought to be associated with IMiD resistance [14–16]. IMiDs selectively bind to CRBN, which mediates the recruitment of Ikaros family zinc finger 1 (IKZF1; Ikaros) or 3 (IKZF3; Aiolos) to E3 ubiquitin ligase for subsequent degradation, resulting in the downregulation of interferon regulatory factor-4 and c-Myc [17]. DNA samples from the bone marrow of IMiD and PI double-refractory MM patients have been examined using next-generation sequencing, and CRBN pathway-related mutations were identified in 32.5% of the patients [18]. Other reports have shown that most patients treated with lenalidomide have downregulated CRBN and upregulated IKZF1 gene expression [19]. CRBN mutations are also associated with pomalidomide resistance in MM [20]. A recent article on whole-genome sequencing (WGS) data from 455 patients and RNA sequencing (RNASeq) data from 655 patients, which included newly diagnosed, lenalidomide-refractory, and pomalidomide-refractory cohorts, reported an increase in the frequency of CRBN aberrations [20]. These aberrations include point mutations, copy losses/structural variations, and a specific variant transcript (exon 10 spliced), with progressive IMiD exposure.

Histone deacetylase (HDAC) inhibitors are known to activate innate immunity [21]. Meanwhile, HDAC inhibition reverses aberrant epigenetic changes contributing to tumor proliferation and enhances tumor immunogenicity [22–24]. HDAC inhibitors also enhance the expression of major histocompatibility complex class I-related chain A and B (MICA and MICB), both of which serve as key natural killer group 2D (NKG2D) ligands [25–27], in leukemic cell lines [28]. NK cells are activated by NKG2D receptor crosslinking on effector cells with NKG2D ligands expressed on tumor cells [25, 29]. In addition to MICA and MICB, NKG2D ligands include UL16-binding proteins (ULBp) [25–27]. Activation of these NKG2D ligands enhances the antibody-dependent cellular cytotoxicity (ADCC) response mounted during mAb therapy for MM [30]. Hence, we proposed a strategy to induce activation of anti-tumor immunity with HDAC inhibitors to overcome resistance in lenalidomide relapse/refractory MM patients.

Moreover, the PI3K/Akt pathway is aberrantly activated in MM [31]. This activation enhances the phosphorylation of glycogen synthase kinase-3 (p-GSK-3) by Akt. Meanwhile, GSK-3 degrades c-Myc, and becomes inactivated by phosphorylation [32, 33]. Hence, increased p-GSK-3 reportedly leads to c-Myc stabilization and MM cell survival [32, 33]. Furthermore, lenalidomide resistance has been attributed to enhanced p-GSK-3 levels [34]. Thus, we also focused on Akt inhibition in lenalidomide-resistant MM patients.

## Methods

### Cell lines and culture condition

Human MM cell lines (RPMI8226, U266, KMS-11, H929, OPM-2, MM1.S, ADR-R/RPMI8226, KMS-11/BTZ, OPM-2/BTZ, H929 CRBN sh#395, H929 CRBN sh#610, OPM-2 CRBN sh#395, OPM-2 CRBN sh#610, OPM-2 CRBN KO, and Dex-R/MM1.S) were cultured in RPMI-1640 medium supplemented with 10% FBS and 1% penicillin/streptomycin. KHYG-1 cells were cultured in RPMI-1640 medium supplemented with 10% FBS and 1% penicillin/streptomycin in the presence of 2–20 ng/mL IL-2. Cell lines were acquired as follows: RPMI8226, U266, H929, and OPM-2 cells were obtained from American Type Culture Collection (ATCC Virginia, USA); KMS-11, KMS-11/BTZ, and KHYG-1 cells were obtained from the Japanese Collection of Research Bioresources Cell Bank (JCRB Osaka, Japan); MM1.S, ADR-R/RPMI8226 and Dex-R/MM1.S cells were kindly provided by Hiroshi Yasui of the University of Tokyo; OPM-2 and OPM-2/BTZ cells were kindly provided by Masaki Ri and Shinsuke Iida of Nagoya City University; OPM-2 CRBN sh#395, OPM-2 CRBN sh#610, and OPM-2 CRBN KO cells were kindly provided by Junichi Yamamoto, Takumi Ito, and Hiroshi Handa of the Tokyo Medical University.

### Reagents

Panobinostat (LBH589), romidepsin, ACY-1215 (ricolinostat), and CUDC-907 were obtained from Selleck Chemicals (Houston, Texas, USA). Each chemical was dissolved in dimethyl sulfoxide (DMSO) and added to the culture medium at the indicated concentrations for in vitro studies. For in vivo experimentation, CUDC-907 was dissolved in DMSO and PEG300, after which Tween80 (polyoxyethylene sorbitan monooleate) and sterile water were added (CUDC-907 5%, PEG300 40%, Tween80 5%, water 50%). Daratumumab for in vitro studies was kindly provided by Janssen Pharmaceutical K. K (Beerse, Antwerpen, Belgium). Elotuzumab was obtained from Bristol-Myers Squibb (New York, New York, USA). Daratumumab and elotuzumab were dissolved in sterile water. CHIR-99021 was obtained from Cayman Chemical Company (Ann Arbor, Michigan, USA).

### Western blot analysis and immunoprecipitation

For the preparation of cell lysates, collected cells were washed with PBS and lysed in radio-immunoprecipitation assay (RIPA) buffer containing dithiothreitol, ethylene glycol-bis(2-aminoethylether)-N, N, N′, N′-tetraacetic acid (EGTA), protease inhibitors, and phenylmethylsulfonyl fluoride (PMSF). Cell lysates were incubated for 5 min at 4 °C, gently shaken for 30 min at 4 °C, and centrifuged at 20000×*g* for 10 min. The supernatant was collected and used for SDS-PAGE analysis. Western blotting was performed using anti-caspase-3 (Cell Signaling Technology, Danvers, Massachusetts, USA. #9662), anti-cleaved caspase-3 (Cell Signaling Technology, #9661), anti-caspase-7 (Cell Signaling Technology, #9494), anti-cleaved caspase-7 (Cell Signaling Technology, #9491), anti-Ikaros (Cell Signaling Technology, #5443), and anti-Actin (Sigma-Aldrich, St. Louis, Missouri, USA. A4700-2ML) primary antibodies. Primary antibody labeling was detected using Immobilon Forte Western HRP substrate (Merck Millipore, Burlington, Massachusetts, USA). Images were analyzed with a LAS-4000 mini (FUJIFILM, Tokyo, Japan). All western blotting assays were repeated at least twice.

### Flow cytometry

MM cell lines (RPMI8226, U266) were exposed to HDAC inhibitors (panobinostat, romidepsin, ACY-1215, CUDC-907) for 24 h. Then, the cell lines were harvested, and the changes in MICA/B, ULBp-2/5/6, and CD38 expression were analyzed by flow cytometry using a BD FACS Verse flow cytometer (BD Biosciences, East Rutherford, New Jersey, USA). MM cells were stained with PE anti-human MICA/B (BioLegend, San Diego, California, USA: 320906), anti-hULBP-2/5/6 (R&D systems, Minneapolis, Minnesota, USA: FAB 1298P), and PE anti-human CD38 (BioLegend: 356604) antibodies. Changes in the expression of markers were assessed by determining the ratio of mean fluorescence intensity (MFI) of MICA/B, ULBp-2/5/6, and CD38, between HDAC inhibitor-exposed cell lines and DMSO-exposed cell lines. This experiment was repeated five times.

### Quantitative reverse transcription PCR (qRT-PCR)

MM cell lines (RPMI8226, U266) were exposed to HDAC inhibitors (ACY-1215, CUDC-907, panobinostat, and romidepsin) and control agents for 0 h, 1 h, 2 h, or 4 h. After treatment, total RNA was extracted from MM cells using an RNeasy Mini Kit (Qiagen, Hilden, Germany. 74,104). cDNA was synthesized from total RNA using the SuperScript III First-Strand Synthesis System (Thermo Fisher, Waltham, Massachusetts, USA. 18,080,051). Real-time PCR was performed for mRNA obtained from MM cell lines. RNA extraction, cDNA synthesis, and qRT-PCR were performed, as previously reported [35]. All qRT-PCR assays were performed in triplicate and repeated at least twice. The expression of MICA, MICB, IKZF1, IKZF3, and Myc in MM cell lines was normalized to 18S ribosomal RNA expression. The expression levels of MICA, MICB, IKZF1, IKZF3, and Myc after each hour of HDAC inhibitor administration were divided by the expression levels of MICA, MICB, IKZF1, IKZF3, and Myc after each hour of control drug administration and expressed as a ratio. qRT-PCR experiments were performed using TaqMan Universal Master Mix II, no UNG (Thermo Fisher), and CFX Connect Real-Time PCR Detection System (Hercules, Bio-Rad, California, USA). The following primers were used: TaqMan Gene Expression Assays (Thermo Fisher Scientific, Massachusetts, USA) for MICA (Hs.130838), MICB (Hs.731446), IKZF1 (Hs.435949), IKZF3 (Hs.4351372), Myc (Hs.4331182), and 18S (Hs.99999901). The details of the primer sequences were not provided by the company.

### ADCC assay and NK assay

Luciferase-expressing MM cell lines were exposed to HDAC inhibitor or DMSO for 24 h. MM cells were then treated with 0.001, 0.01, 0.1, 1, or 10 μg/mL daratumumab, elotuzumab, or control (IgG) and co-incubated with NK cells. NK cells were extracted from PBMCs obtained from healthy donors using a human NK Cell Isolation Kit (Miltenyi Biotec, Nordrhein-Westfalen, Germany). Cell counting was performed with a hemocytometer (Erma Inc., Tokyo, Japan). NK cells were collected in RPMI-1640 with 10% FBS and 1% penicillin/streptomycin. Fresh NK cells were added at a ratio of 10:1 to MM cells. Cell death was calculated from the decrease in luciferase activity, which was detected by Steady Glo (Promega, Madison, Wisconsin, USA) or PicaGene (TOYO INK, Tokyo, Japan). Luciferase luminescence in the samples was evaluated using a Nivo spectrophotometer (Perkin Elmer, Massachusetts, USA). ADCC and NK assays were repeated at least twice.

### Methyl thiazolyl tetrazolium assay

Each MM cell line was seeded in a 96-well plate and incubated with HDAC inhibitors, Akt inhibitor, GSK-3 inhibitor, and PI for 48–72 h. Cells were exposed to lenalidomide and pomalidomide for 5 days, doxorubicin for 5 h, or dexamethasone for 1 week. Methyl thiazolyl tetrazolium (MTT) assays were performed after staining with a Cell Counting Kit-8 (Dojindo Laboratories, Kumamoto, Japan) for 50 min. Plates were read using a Nivo spectrophotometer. Each assay was performed in quintuplicates, and the experiment was repeated at least twice.

### In vivo animal experiments

Six-week-old C.B-17/Icr-*scid*/*scid*Jcl mice were purchased from Japan CLEA (Tokyo, Japan). Mice were subcutaneously injected with OPM-2 CRBN KO (OPM-2 CRBN-knockout cells) to generate SCID mice. After tumor cell injection, SCID mice with tumors over 10 mm in length along the major axis were treated with vehicle or CUDC-907 (50 mg/kg body weight) three times per week for 2 weeks. For the experiments, eight mice per study group (vehicle and CUDC-907 treated) were used. The outcome was a change in tumor size compared to the day treatment started. Mice were observed for 14 days after administration. This animal experiment was approved by the Animal Experiment Committee at the Institute of Medical Science of the University of Tokyo (see Study approval). The description was based on The ARRIVE guidelines 2.0 [36].

### Immunohistochemistry

Tumors of OPM-2 CRBN KO (CRBN knockout cells) were isolated when the SCID mice were sacrificed 13 days after starting the treatment with the vehicle or CUDC-907 (the next day after all oral administrations are completed). Formalin-fixed paraffin-embedded sections (3 μm) were deparaffinized and hydrated with graded ethanol. After heat-induced antigen retrieval for 10 min at 120 °C, slides were incubated in the following primary antibodies: a 1:300 dilution of cleaved caspase-3 rabbit polyclonal antibody (Cell Signaling Technology, #9661, Massachusetts, USA), a 1:200 dilution of GSK3 beta (phospho Y216) + GSK3 alpha (phospho Y279) rabbit polyclonal antibody (Abcam, ab75745, Cambridge, UK), a 1:100 dilution of c-Myc rabbit monoclonal antibody (clone Y69, Abcam, ab32072) for 30 min at 37 °C respectively. Labeling of secondary antibody was performed with Histofine Simple Stain Mouse MAX-PO(R) (Nichirei Bioscience, Tokyo, Japan) for 30 min at 37 °C. To visualize the antigen-antibody complex, the ImmPACT DAB substrate kit (Vector Laboratories, Burlingame, CA, USA) was used, and then sections were counterstained with hematoxylin. A section from a breast cancer tissue placed on the same slide glass was used as the positive control, and sections treated with PBS instead of the primary antibodies were used as negative controls.

### In vitro experiments using patient samples or healthy donors

Bone marrow samples were collected from MM patients (newly diagnosed or relapsed/refractory myeloma). The primary cells used were derived from 5 patients with newly diagnosed MM and seven patients with relapsed/refractory MM. Upon patient consent, an additional 5 mL of bone marrow fluid was collected during a routine bone marrow examination. MM cells were then sorted using FACS Aria (BD Biosciences). The primary cells were cultured in RPMI-1640 medium supplemented with 10% FBS and 1% penicillin/streptomycin. For the control sample, 20 mL peripheral blood was collected from healthy donors after obtaining consent. Then, peripheral blood mononuclear cells (PBMNCs) were isolated using Lymphoprep (Cosmo Bio, Tokyo, Japan) and cultured in RPMI-1640 medium supplemented with 10% FBS and 1% penicillin/streptomycin and 2 μg/mL phytohemagglutinin-L (PHA-L; Sigma-Aldrich, Missouri, USA).

### Statistics

For each analysis, *P*-values < 0.05 were considered to represent statistically significant differences.

For the FACS experiments, the expression level relative to DMSO were tested by one-sample t-test with null hypothesis of the ratio = 1. As for some multiple comparisons, we performed ANOVA with post hoc Dunnett’s test for the data from the qRT-PCR, and Kruskal-Wallis followed by Mann-Whitney with Bonferroni correction for MTT assays. Statistical methods were selected according to the data distributions assessed by appearance in the plots. Synergistic effects were evaluated by a generalized linear model including the statistical interaction term of two drugs (distribution: normal. Link function: log). Effects on ADCC activity and cell viability were evaluated by a general linear regression model with the statistical interaction term of drugs and log-transformed dose or effector/target (E/T) ratio. Repeated measurements in mice were evaluated by a linear mixed regression model with the statistical interaction term of drugs and time (days), considering intra-individual correlation by including random effects. *P*-values were calculated for the interaction terms in the regression models, which were interpreted as the difference in slope of dose−/time-dependent change between groups.

## Results

### HDAC inhibitors upregulate NKG2D ligands and enhance mAb effects in MM cell lines

**Fig. 1:**
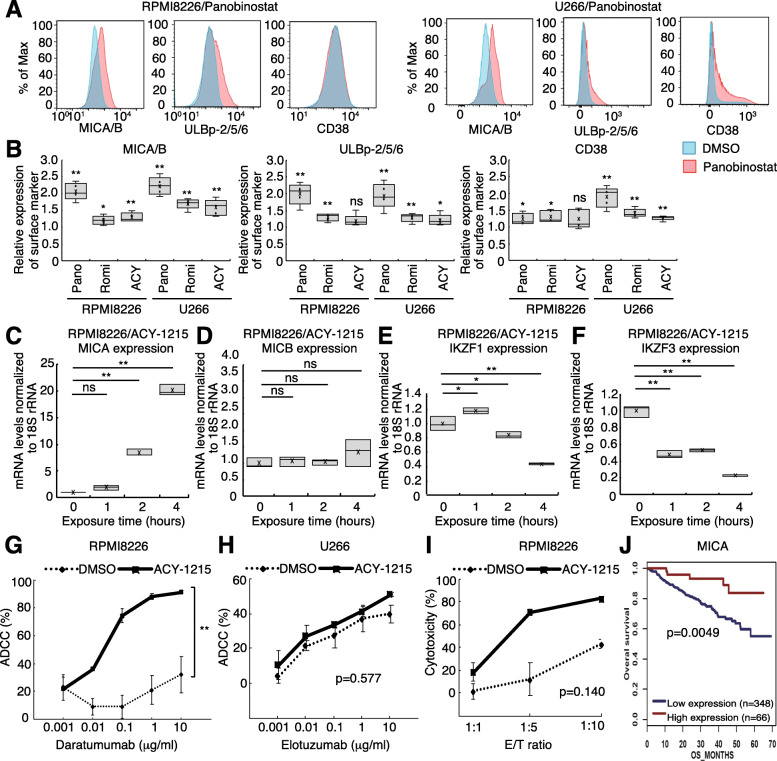
HDAC inhibitors enhance NK cell activity via NKG2D ligand upregulation and IKZF1/3 downregulation. **a** Histograms of MICA/B, ULBp-2/5/6, and CD38 expression in RPMI8226 and U266 cells treated with panobinostat for 24 h. **b** Summary of the ratio of mean fluorescence intensity (MFI) for MICA/B, ULBp-2/5/6, and CD38 expression between cells exposed to HDAC inhibitors [Panobinostat (Pano), Romidepsin (Romi), ACY-1215 (ACY)]. The MFI values of MICA/B, ULBp-2/5/6, and CD38 of MM cells upon exposure to each HDAC inhibitor were divided by the corresponding MFI values upon exposure to DMSO. Experiments were repeated 5 times. Error bars represent the SD. (*p < 0.05, **p < 0.01, “ns” indicates no significant difference). **c–f** MICA, MICB, IKZF1/3 mRNA levels in RPMI8226 cells exposed to ACY-1215. Experiments were performed in triplicate (*n* = 3). (*p < 0.05, “ns” indicates no significant difference). **g–i** ADCC and NK cell assay of daratumumab and elotuzumab in the presence of ACY-1215. Experiments were repeated three times (*n* = 3). Error bars represent the SD. (**p < 0.01) **j** Prognostic value of MICA expression based on MM patient clinical trial data

In a previous study, HDAC inhibitors enhanced NKG2D ligand expression in hepatoma cells and sensitized them to NK-mediated cytolysis [37]. Panobinostat reportedly increased CD38 expression in MM cells in a time-dependent manner, enhancing the efficacy of daratumumab [38]. However, it is unknown whether panobinostat alters NKG2D ligand expression on MM cells. We found that 24 h panobinostat treatment increased NKG2D ligand (MICA/B and ULBp-2/5/6) expression in RPMI8226 and U266 cells. Panobinostat increased CD38 expression in both RPMI8226 and U266, but only slightly in RPMI8226 (Fig. 1a). Romidepsin enhanced surface marker MICA/B, ULBp-2/5/6, and CD38 expression in RPMI8226 and U266. ACY-1215 enhanced MICA/B expression of RPMI8226 and MICA/B, ULBp-2/5/6, and CD38 expression in U266 (Fig. 1b). Next, we tested whether MICA and MICB expression is altered under HDAC inhibition at the mRNA level. Panobinostat and ACY-1215 increased MICA mRNA expression in RPMI8226 and U266 cells (Fig. 1c, Supplementary Fig. 1A-C). MICB expression did not significantly increase after ACY-1215 treatment in RPMI8226 (Fig. 1d). Panobinostat slightly enhanced MICB in U266 cells (Supplementary Fig. 1D). Other results are summarized in Supplementary Fig. 1E.

IKZF1 and IKZF3 are negative regulators of MICA [39]. We found that ACY-1215 significantly downregulated IKZF1 (Fig. 1e) and IKZF3 (Fig. 1f) at the mRNA level after 4 h exposure. Moreover, panobinostat significantly downregulated IKZF1 in U266 cells (Supplementary Fig. 1F).

As it has been suggested that HDAC inhibitors may act on NKG2D ligands of MM cells, we decided to investigate how the combined use of HDAC inhibitors on mAbs against MM cells affects ADCC activity. Based on previous reports [40, 41], we aimed to verify the ADCC activity of daratumumab in RPMI8226 cells and that of elotuzumab in U266. We found that daratumumab ADCC was significantly upregulated by ACY-1215 treatment (Fig. 1g). Additionally, ACY-1215 tended to enhance the ADCC activity of elotuzumab (Fig. 1h) and the direct cytotoxicity of NK cells against MM cells (Fig. 1i).

Analysis of gene expression profiles from 414 newly diagnosed MM patients [42] deposited in an integrated gene expression and disease prognosis database (GenomicScape, http://www.genomicscape.com) revealed that higher MICA expression was significantly associated with better overall survival (OS; *p* = 0.0049, Fig. 1j). We also analyzed data from a clinical trial that compared the efficacy and safety of bortezomib treatment [43]. The data indicated longer OS of patients with high MICA and ULBp2 expression compared with that of patients with low MICA (*p* = 0.068, Supplementary Fig. 1G) and ULBp2 (*p* = 3.6e-05, Supplementary Fig. 1H) expression. We hypothesized that upregulation of NKG2D ligands, including MICA and ULBp2, is critical for enhanced MM cell recognition by NK cells and contributes to better clinical outcomes.

### HDAC inhibitors upregulate NKG2D ligands independently of CRBN expression

**Fig. 2:**
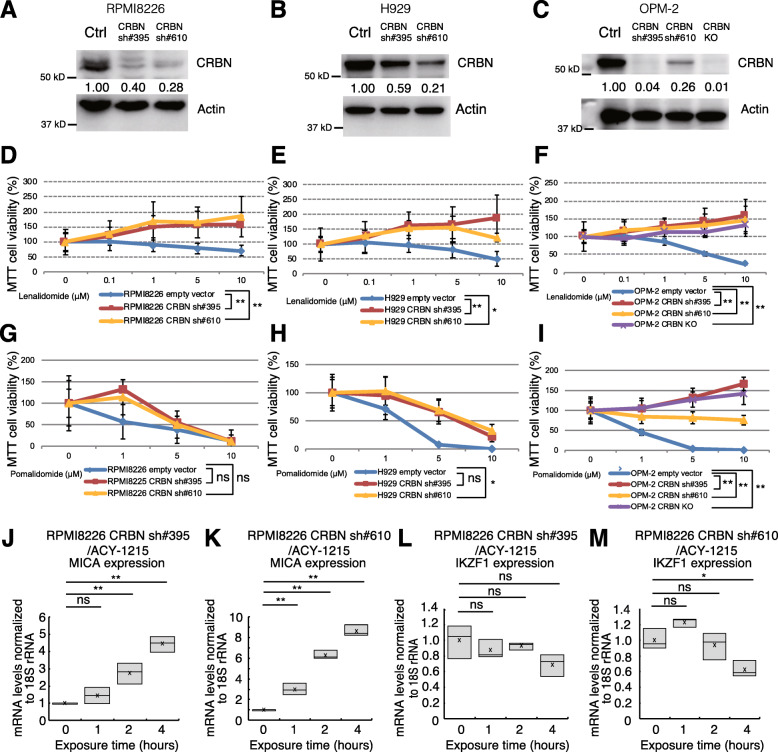
HDAC inhibitors enhance NKG2D ligand expression via a CRBN-independent pathway. **a–c** CRBN protein levels in RPMI8226, H929, and OPM-2 cells. The number below the band shows the density of CRBN normalized to that of actin. **d–i** MTT proliferation assay. Sensitivity of CRBN knockdown and CRBN-knockout MM cell lines to lenalidomide and pomalidomide. DMSO vehicle control and the corresponding values of MTT cell viability were designated as 100% (0 μM). To show whether inhibitors compromised cell proliferation of CRBN-knockdown cells, the ratio (%) of MTT cell viability for each concentration of lenalidomide or pomalidomide used, compared with DMSO vehicle control, is indicated. Each experiment was performed in quintuplicate (*n* = 5). Results are shown as the mean ± SD. (*p < 0.05, **p < 0.01, “ns” indicates no significant difference). **j-m** MICA and IKZF1 mRNA levels in CRBN-deficient RPMI8226 cells exposed to ACY-1215. Experiments were performed in triplicate (*n* = 3). (*p < 0.05, **p < 0.01, “ns” indicates no significant difference)

We established CRBN-deficient RPMI8226 (Fig. 2a), H929 (Fig. 2b), and OPM-2 cells (Fig. 2c) by RNAi-mediated downregulation of CRBN or CRISPR-Cas9-mediated CRBN knockout to establish lenalidomide-resistant cells. The CRBN-deficient cell lines were experimentally validated using an MTT proliferation assay. CRBN-deficient cells were resistant to lenalidomide cytotoxicity, proliferating under high lenalidomide concentrations (Fig. 2d–f). In contrast, pomalidomide cytotoxicity [44] was observed in both CRBN-knockdown and parent RPMI8226 cells (Fig. 2g).

The TP53RK-mediated Myc inhibitory pathway is activated by pomalidomide but not by lenalidomide [45]. Pomalidomide was speculated to suppress Myc in CRBN-knockdown cells, not via the CRBN-mediated pathway, rather via the TP53RK-mediated pathway. However, decreased pomalidomide cytotoxicity was observed in CRBN-deficient H929 CRBN sh#610 cells when compared to their CRBN-intact counterparts (Fig. 2h). For OPM-2 cells, pomalidomide cytotoxicity decreased with lower CRBN expression (Fig. 2i). Therefore, pomalidomide was not effective against all lenalidomide-resistant cells.

Next, we investigated whether HDAC inhibitors were able to overcome IMiD resistance in CRBN-deficient MM cells. ACY-1215 treatment of CRBN-deficient cells resulted in the upregulation of MICA mRNA (Fig. 2j-k). Furthermore, ACY-1215 treatment tended to reduce IKZF1 mRNA in RPMI8226 CRBN sh#395 (Fig. 2l). ACY-1215 significantly downregulated IKZF1 in RPMI8226 CRBN sh#610 (Fig. 2m). These observations suggest that ACY-1215-dependent IKZF1 downregulation and MICA upregulation occur via a CRBN-independent pathway, as well as that HDAC inhibitors can overcome CRBN-associated IMiD resistance through IKZF1 downregulation.

### HDAC and Akt inhibitors are potential treatments for lenalidomide-resistant cells

**Fig. 3:**
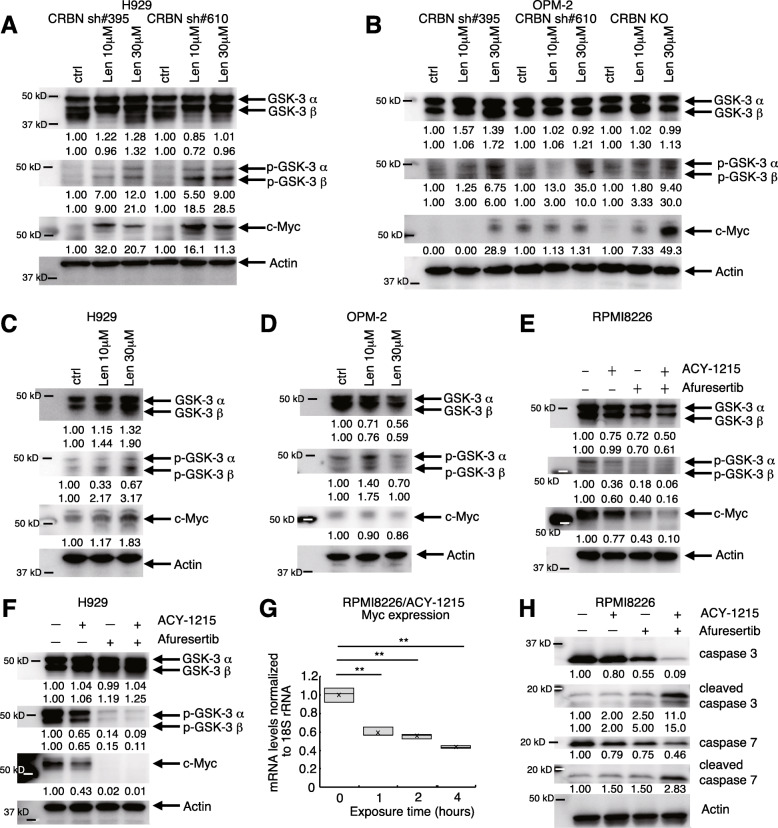
HDAC and Akt inhibitor treatment can overcome c-Myc upregulation by lenalidomide in CRBN-deficient cells. **a-b** Western blot validation of the effects of lenalidomide (Len) on CRBN-deficient cells. c-Myc expression could not be detected in OPM-2 CRBN sh#395 cells exposed to the control drug and Len 10 μM. **c-d** Validation of the effects of Len on control H929 or OPM-2 cells via western blot analysis. **e-f** RPMI8226 and H929 cell lysates treated with ACY-1215 and/or afuresertib were immunoblotted with the indicated antibodies (against GSK-3, p-GSK-3, c-Myc, and actin). **g** Myc mRNA levels in RPMI8226 cells treated with ACY-1215. (**p < 0.01) **h** RPMI8226 cell lysates treated with ACY-1215 and/or afuresertib were immunoblotted with indicated antibodies (against caspase-3, cleaved caspase-3, caspase-7, cleaved caspase-7, actin)

Enhanced p-GSK-3 α/β expression was previously reported in MM cell lines following extended exposure to lenalidomide, in turn compromising lenalidomide sensitivity [34]. Thus, we examined how treatment with 10 and 30 μM lenalidomide for 1 month would alter p-GSK-3 α/β levels [34] in H929 and OPM-2 cells when CRBN was downregulated to a variable extent. We observed a phosphorylation-dependent mobility shift of GSK-3 α/β and an increase in p-GSK-3 α/β and c-Myc levels in H929 cells after lenalidomide exposure (Fig. 3a). Increased p-GSK-3 α/β and c-Myc levels were also observed in OPM-2 cells (Fig. 3b). These changes were exacerbated when cells were exposed to higher lenalidomide concentrations, implying that CRBN disruption in MM cells could further increase c-Myc levels after extended lenalidomide treatment. Previous report showed that CRBN-deficient activated mouse CD8^+^ T cells and human CD8^+^ T cells treated with immune-modulating compounds exhibited increased Myc levels [46]. The mechanism behind this is unknown, but it is possible that a similar reaction was caused by lenalidomide in MM cells. We also exposed control H929 and OPM-2 cells to 10 or 30 μM lenalidomide, but only for 5 days as both concentrations were shown to kill the cells (Fig. 2e-f). In control H929, p-GSK-3 β and c-Myc was increased (Fig. 3c), however, p-GSK-3 and c-Myc expression was not significantly increased in control OPM-2 cells (Fig. 3d). These results indicate that the increase in p-GSK3 α/β and c-Myc due to lenalidomide treatment was more prominent in CRBN-low cells than in control cells.

Since GSK-3 and c-Myc are located downstream of the PI3K/Akt pathway, it was speculated that Akt inhibitors might also be useful for MM therapy. We, therefore, assessed the effects of HDAC inhibitors, Akt inhibitors, and the combination of HDAC inhibitors and Akt inhibitors. Oral Akt inhibitor afuresertib has been clinically tested in patients with advanced MM. Both phase I [47] and II [48] trials of afuresertib were performed in relapsed/refractory malignant lymphoma patients. However, the efficacy of combined HDAC and Akt inhibitor treatment in MM is currently unknown. We measured GSK-3 α/β, p-GSK-3 α/β, and c-Myc protein levels following HDAC inhibitor and/or Akt inhibitor treatment. ACY-1215 and/or afuresertib decreased p-GSK-3 α/β and c-Myc protein levels in RPMI8226 and H929 cells (Fig. 3e-f), suggesting that HDAC inhibitors and Akt inhibitors may overcome lenalidomide-enhanced c-Myc expression in CRBN-deficient cells. The downregulation of p-GSK-3 and c-Myc appeared to be a consequence of downregulation in Akt expression caused by the ACY-1215 (Supplementary Fig. 2A). ACY-1215 also downregulated c-Myc mRNA in RPMI8226 cells (Fig. 3g). According to clinical trial data comparing the efficacy and safety of treatment with and without bortezomib [43], patients with high GSK-3 α/β expression had better OS than those with low GSK-3 α (*p* = 1.9 × 10^− 9^, Supplementary Fig. 2B)/β (*p* = 0.02, Supplementary Fig. 2C) expression. Further, patients with high Myc expression had poorer OS than those with low expression (*p* = 0.0043, Supplementary Fig. 2D). These results are suggestive of the negative effect of GSK-3 α/β inactivation-related c-Myc stabilization on MM patient prognosis.

Finally, we investigated the proapoptotic effect of ACY-1215 and afuresertib alone and in combination. Both inhibitors induced apoptosis, and combined treatment had an even stronger effect (Fig. 3h, Supplementary Fig. 2E).

### HDAC and Akt inhibitors exhibit cytotoxicity in several MM lines, including drug-resistant MM cell lines

**Fig. 4:**
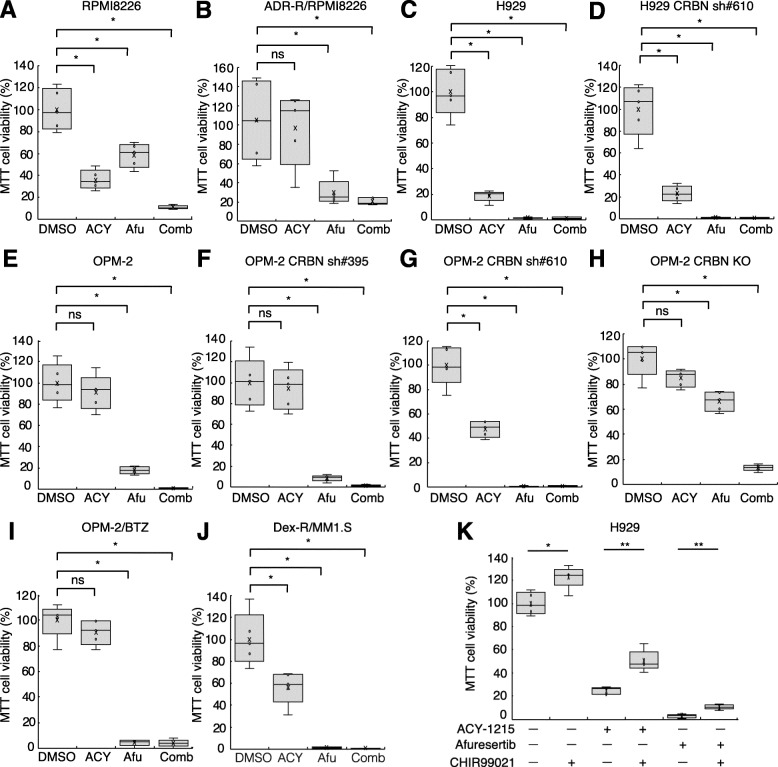
HDAC and Akt inhibitors exhibit cytotoxicity in several MM cell lines. **a-j** MTT proliferation assay. Each cell line was treated with DMSO, 2 μM ACY-1215 (ACY), 4 μM afuresertib (Afu), or a combination of ACY-1215 and afuresertib (Comb) for 72 h. The average was calculated for each experiment performed in quintuplicate (*n* = 5). (*p < 0.05, **p < 0.01, “ns” indicates no significant difference). **k** H929 were treated with 2 μM ACY-1215, 4 μM afuresertib with/without 1 μM CHIR 99021 (GSK-3 inhibitor) for 48 h. Experiments were performed in quintuplicate (*n* = 5). (**p < 0.01, ***p < 0.001)

Since HDAC inhibitors and Akt inhibitors were found to induce apoptosis in MM cells, we decided to confirm their cytotoxicity in MM cells by MTT assay. Several MM lines, including drug-resistant cells, received ACY-1215 and/or afuresertib. Combined treatment exerted greater cytotoxic effects on RPMI8226 cells than each inhibitor alone (Fig. 4a). Similar results were observed in doxorubicin-resistant RPMI8226 (Fig. 4b), U266 (Supplementary Fig. 2F), H929 (Fig. 4c), CRBN-deficient H929 (Fig. 4d and Supplementary Fig. 2G), OPM-2 (Fig. 4e), CRBN-deficient OPM-2 (Fig. 4f-h), bortezomib-resistant OPM-2 (Fig. 4i), KMS-11 (Supplementary Fig. 2H), bortezomib-resistant KMS-11 (Supplementary Fig. 2I), MM1.S (Supplementary Fig. 2 J), and dexamethasone-resistant MM1.S (Fig. 4j). The synergistic effects of ACY-1215 and afuresertib were observed in RPMI8226, U266, KMS-11, OPM-2, OPM-2 CRBN sh#395, and OPM-2 CRBN sh#610 cells. We then evaluated whether GSK-3 inhibition impairs the anti-MM activity of HDAC and Akt inhibitors by using GSK-3 inhibitor CHIR 99021 [49]. The use of CHIR 99021 with ACY-1215 or afuresertib reduced the cytotoxicity of ACY-1215 and afuresertib. However, this was not enough to completely eliminate ACY-1215 and afuresertib cytotoxicity (Fig. 4k), suggesting that anti-MM effects of ACY-1215 and afuresertib were partly mediated via GSK-3 activation.

### Dual HDAC and PI3K inhibitor CUDC-907 upregulates NKG2D ligands, enhancing daratumumab and elotuzumab activity

**Fig. 5:**
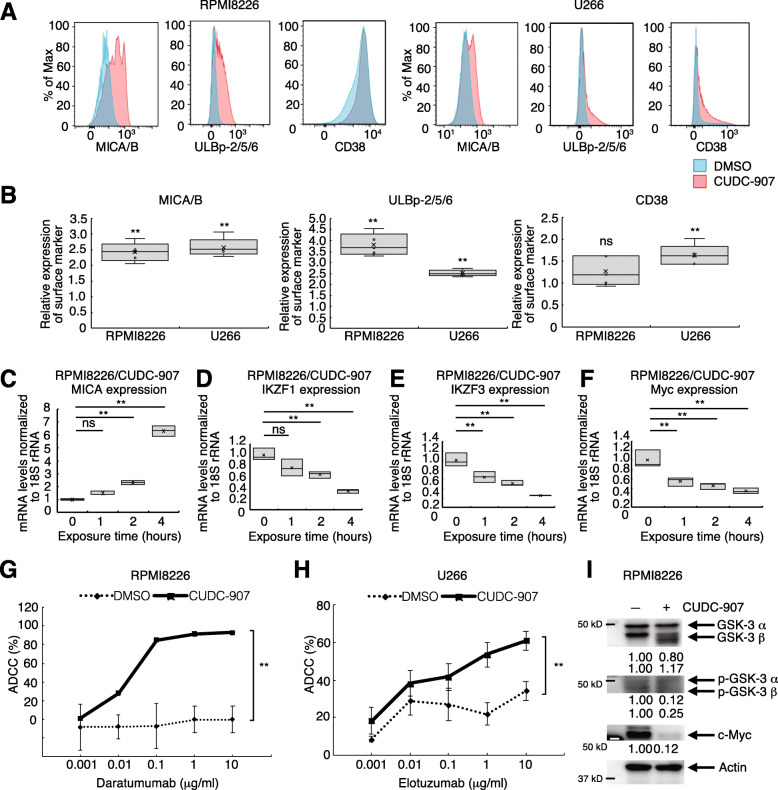
CUDC-907 enhances ADCC of mAbs by upregulating MICA, ULBp-2/5/6, CD38 while downregulating c-Myc. **a** Histograms show MICA/B, ULBp-2/5/6, and CD38 expression in RPMI8226 and U266 cells treated with 10 nM CUDC-907. **b** Summary of the ratio of MFI for MICA/B, ULBp-2/5/6, and CD38 expression in cells exposed to CUDC-907. The MFI values of MICA/B, ULBp-2/5/6, and CD38 of MM cells upon exposure to CUDC-907 were divided by the corresponding MFI values upon exposure to DMSO. Experiments were repeated 5 times. Error bars represent the SD. (*p < 0.05, **p < 0.01, “ns” indicates no significant difference). **c-f** MICA, IKZF1/3, Myc mRNA levels in RPMI8226 cells exposed to CUDC-907. Experiments were performed in triplicate (*n* = 3). (*p < 0.05, **p < 0.01, “ns” indicates no significant difference). **g–h** ADCC assay of daratumumab and elotuzumab. Experiments were performed in triplicate (*n* = 3), and data are presented as the mean ± SD. *P*-values were calculated for the interaction terms in the regression models, which are interpreted as the difference in slope of dose-dependent change between the groups (**p < 0.01). **i** Western blot validation of the effects of CUDC-907 on RPMI8226 cells

CUDC-907 is a dual HDAC and PI3K inhibitor [50, 51] that is currently being evaluated in clinical trials for the treatment of MM [52] and lymphoma [53]. CUDC-907 inhibits PI3K, which is an upstream activator of Akt in the PI3K/Akt pathway [33]. We found that CUDC-907 upregulated MICA/B and ULBp-2/5/6 expression in RPMI8226 and U266. CUDC-907 did not increase the expression of CD38 in RPMI8226 but increased it in U266 (Fig. 5a-b). CUDC-907 also upregulated MICA mRNA (Fig. 5c) and downregulated IKZF1 (Fig. 5d), IKZF3 (Fig. 5e), and Myc (Fig. 5f) mRNA levels in RPMI8226 cells. CUDC-907 significantly enhanced the ADCC activity of daratumumab (Fig. 5g) and elotuzumab (Fig. 5h), while also downregulating p-GSK-3 α/β and c-Myc expression (Fig. 5i).

### CUDC-907 upregulates NKG2D ligands and exhibits cytotoxicity in CRBN-knockout MM cells

**Fig. 6:**
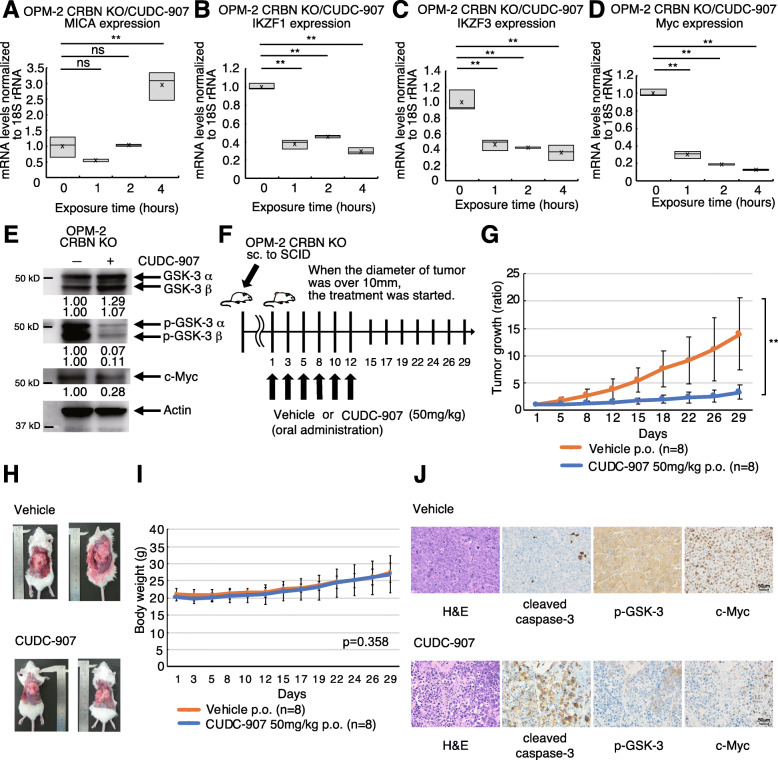
CUDC-907-induced NKG2D ligand upregulation and IKZF1/3 downregulation reduces CRBN-knockout cell growth in vivo. **a–d** mRNA levels of MICA, IKZF1, IKZF3, and Myc in CRBN-knockout OPM-2 cells treated with CUDC-907. Experiments were performed in triplicate (*n* = 3). (**p < 0.01, “ns” indicates no significant difference.) **e** OPM-2 CRBN-knockout cell lysate treated with CUDC-907 was immunoblotted with the indicated antibodies. **f** Xenograft mouse model using CRBN-knockout OPM-2 cells. Eight mice were orally administered vehicle (*n* = 8) or 50 mg/kg CUDC-907 (*n* = 8) three times a week for 2 weeks. **g** Tumor growth (ratio) of CRBN-knockout OPM-2 cells in SCID mice treated with either vehicle or CUDC-907. P-values were calculated for the interaction terms in the regression models (**p < 0.01). **h** The representative images of tumors of each group at day 29. **i** Body weight was measured on the indicated days. *P*-values were calculated for the interaction terms in the regression models. **j** Representative patterns of immunohistochemical staining of tumor from SCID mice. Hematoxylin and eosin (H&E) staining and immunohistochemical staining with antibodies specific for cleaved caspase-3, p-GSK-3, and c-Myc showed brown-stained cells (positive) and blue-stained cells (negative). Low cleaved caspase-3 expression in case with vehicle-administered group and high cleaved caspase-3 expression in case with CUDC-907-administered group. Weak expression of p-GSK-3 and c-Myc was observed in the case with CUDC-907-administered group (original magnification × 400)

CUDC-907 treatment upregulated MICA mRNA expression (Fig. 6a) and downregulated IKZF1 (Fig. 6b), IKZF3 (Fig. 6c), and Myc (Fig. 6d) mRNA expression in CRBN*-*knockout MM cells. Further, CUDC-907 suppressed p-GSK-3 α/β and c-Myc expression in CRBN*-*knockout cells (Fig. 6e). We performed xenograft mouse model experiments using CRBN-deficient cells resistant to lenalidomide. Mice (*n* = 8 per group) received vehicle or CUDC-907 (50 mg/kg body weight) orally (Fig. 6f). CUDC-907-treated mice exhibited reduced tumor growth rates compared to vehicle mice (Fig. 6g-h). There was no significant difference in the changes in body weight between vehicle- and CUDC-907-treated mice (*p* = 0.358, Fig. 6i). We analyzed the basic histological and relevant biomarkers of tumors in both CUDC-907 administrated and control groups. Tumors in the CUDC-907 group showed increased apoptosis, which localized with regions showing high expression of cleaved caspase-3. Expression of p-GSK-3 and c-Myc was lower in the CUDC-907 group compared to that in the vehicle group (Fig. 6j).

### Efficacy of CUDC-907 in primary cells and drug-resistant MM cells

**Fig. 7:**
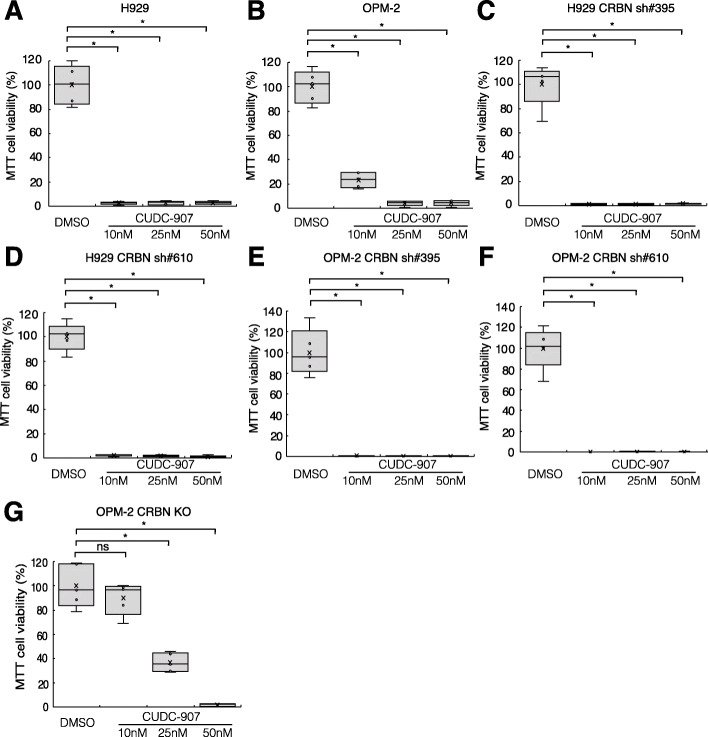
MTT proliferation assay in MM cell lines **a** H929, **b** OPM-2, **c** H929 CRBN sh#395, **d** H929 CRBN sh#610, **e** OPM-2 CRBN sh#395, **f** OPM-2 CRBN sh#610, and **g** OPM-2 CRBN KO cells treated with CUDC-907. Each cell line was treated with DMSO, 10 nM CUDC-907, 25 nM CUDC-907, or 50 nM CUDC-907 for 72 h. The average was calculated for each experiment performed in quintuplicate (*n* = 5). (*p < 0.05, **p < 0.01, “ns” indicates no significant difference)

**Fig. 8:**
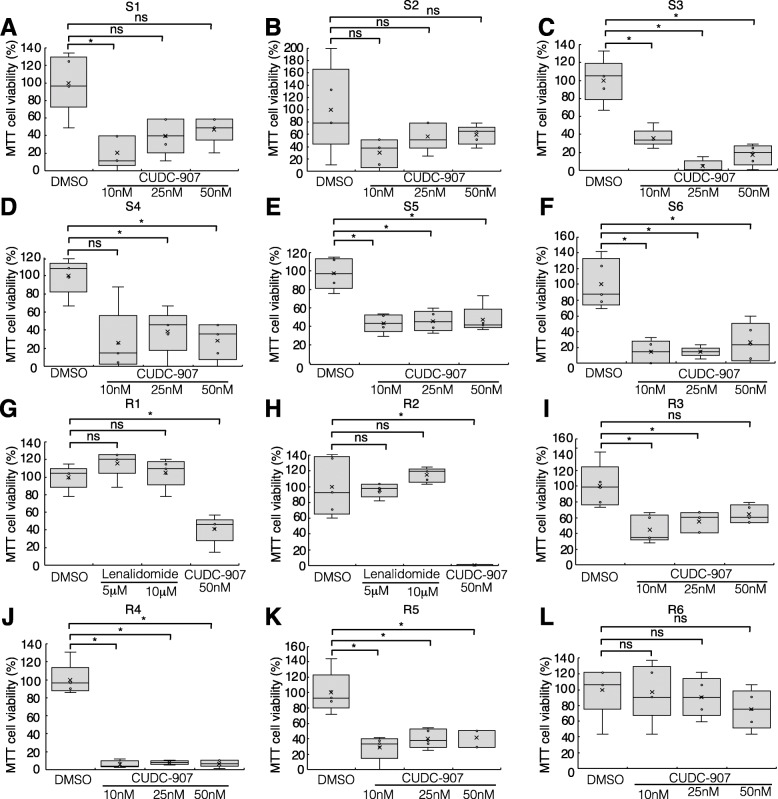
MTT proliferation assay were performed in primary cells from 12 MM patients. **a–f** MTT proliferation assays were performed in primary cells from six lenalidomide-sensitive MM patients. Cells were treated with 10–50 nM CUDC-907 for 48 h. **g-l** MTT proliferation assays were performed in primary cells from six lenalidomide-resistant MM patients. Cells were treated with 5 μM lenalidomide, 10 μM lenalidomide, or 10–50 nM CUDC-907 for 48 h. (*p < 0.05, **p < 0.01, “ns” indicates no significant difference.) Clinical parameters of MM patients are shown in Table 1

We examined the efficacy of CUDC-907 on parental MM cell lines, in addition to lenalidomide-resistant, doxorubicin-resistant, bortezomib-resistant, and dexamethasone-resistant cells (Fig. 7a-g, Supplementary Fig. 3A-H), as well as in primary cells from MM patients (Table 1) sensitive to lenalidomide (S1–S6) (Fig. 8a-f) and six patients refractory to lenalidomide (R1–R6) (Fig. 8g-l). Refractory patients were defined as patients with disease that had no response while on lenalidomide-based therapy or experienced progression within 60 days of their last therapy, as per The International Myeloma Working Group criteria [54]. Lenalidomide (5 μM and 10 μM) showed no cytotoxicity in primary cells from R1 (Fig. 8g) and R2 (Fig. 8h) patients. Thus, we confirmed that cells derived from R1 and R2 patients were resistant to lenalidomide even in vitro. On the other hand, most cells, including R1 and R2 cells, were sensitive to CUDC-907 after 48 h of treatment (Fig. 8a, c-k). Although, primary cells from Patient S2 and R6 were not significantly affected by CUDC-907 (Fig. 8b, l).

**Table 1:** Clinical parameters of MM patients

Patient	Age	Sex	Type of Ig	Clinical stage			Medical history	Lenalidomide refractory
				D&S	ISS	R-ISS		
S1	68	F	IgA-κ	IIIA	III	III	Newly diagnosed	No
S2	78	M	IgG-λ	IIA	II	II	Newly diagnosed	No
S3	78	M	IgG-κ	IIIA	III	N/A	Newly diagnosed	No
S4	46	M	IgG-λ	IIIA	III	III	Newly diagnosed	No
S5	66	M	IgG-κ	IIA	I	II	Newly diagnosed	No
S6	51	F	IgG-λ	IIA	II	II	CyBorD, HDCY, BD, MEL, autoPBSCT	No
R1	56	M	IgG-κ/ BJP-κ	IA	I	N/A	VRd	Yes
R2	55	F	IgA-λ	IIA	I	II	VRd, VP16, MEL, autoPBSCT, biweeklyBor, LEN maintenance, DRd, VTDPACE, DCEP, KRd	Yes
R3	80	M	IgA-κ	IIA	I	N/A	CyBorD, Bor biweekly, VTd, VRd, HDCY, MEL, auto PBSCT	Yes
R4	73	F	BJP-λ	IIIB	III	III	Ld, Eld, DVd, PCd	Yes
R5	62	M	IgG-κ	IIIA	III	II	BD, VRd	Yes
R6	76	M	IgA-λ	IIIA	II	II	CyBorD, VRd, LEN maintenance, DVd, PCd, KRd, Kd	Yes

CyBorD; cyclophosphamide, bortezomib, dexamethasone. HDCY; High dose cyclophosphamide. BD; bortezomib, dexamethasone. MEL; melphalan. PBSCT; peripheral blood stem cell transplantation. VRd; bortezomib, lenalidomide, dexamethasone. VP-16; etoposide. Bor; bortezomib. LEN; lenalidomide. DRd; daratumumab, lenalidomide, dexamethasone. VTD-PACE; bortezomib, thalidomide, dexamethasone, cisplatin, doxorubicin, cyclophosphamide, etoposide. DCEP; dexamethasone, cyclophosphamide, etoposide, cisplatin. KRd; carfilzomib, lenalidomide, dexamethasone. VTd; bortezomib, thalidomide, dexamethasone. Ld; lenalidomide, dexamethasone. Eld; elotuzumab, lenalidomide, dexamethasone. DVd; daratumumab, bortezomib, dexamethasone. PCd; pomalidomide, cyclophosphamide, dexamethasone. Kd; carfilzomib, dexamethasone. D&S:Durie-Salmon staging. ISS:International Staging System. R-ISS:Revised International Staging System

Finally, we examined how HDAC and Akt inhibitors, as well as CUDC-907, affect PBMNCs of healthy donors to survey normal cellular functions. We found that the HDAC inhibitor, the Akt inhibitor, and CUDC-907 did not show cytotoxicity toward PBMNCs from healthy donors (Supplementary Fig. 3I).

## Discussion

In the current study, we investigated whether small-molecule HDAC and Akt inhibitors could overcome lenalidomide resistance through suppression of tumor growth and enhancement of immunotherapy in MM. HDAC inhibitors reduced tumor growth by downregulating c-Myc in a CRBN-independent manner, while upregulating MICA expression and, thus, enhancing the efficacy of immunotherapy. In particular, HDAC inhibitors enhanced the ADCC activity of daratumumab and elotuzumab.

Lenalidomide is currently used for induction and maintenance therapy in MM treatment. However, the development of lenalidomide resistance worsens the prognosis of MM patients. Thus, overcoming this challenge is important for improving clinical outcomes. CRBN is the primary target of IMiDs [12–14], and low CRBN expression is associated with resistance to lenalidomide and pomalidomide monotherapy [15]. Unlike lenalidomide, pomalidomide exhibited cytotoxicity in some CRBN-knockdown cells.

Surprisingly, CRBN-knockdown and -knockout cells cultured with lenalidomide showed an upregulation of p-GSK-3 and c-Myc. These results are consistent with the significant increase in c-Myc following the development of lenalidomide resistance, when compared to its levels in bone marrow samples collected from MM patients at diagnosis [16]. Our data indicate that c-Myc is an important therapeutic target in CRBN-deficient MM cells.

As GSK-3 and c-Myc are located downstream of the PI3K/Akt pathway, we explored the effects of Akt inhibition. Akt inhibitor afuresertib downregulated p-GSK-3 α/β and c-Myc at the protein level. Furthermore, combined HDAC and Akt inhibitor treatment induced stronger downregulation of c-Myc than either inhibitor alone. Thus, combined HDAC and Akt inhibition could suppress the proliferation of lenalidomide-resistant cell lines by overcoming c-Myc upregulation.

Based on the synergetic suppression of MM cell proliferation by combined HDAC and Akt inhibition, we evaluated dual HDAC and PI3K inhibitor CUDC-907. CUDC-907 enhanced daratumumab and elotuzumab ADCC, while also upregulating NKG2D ligand expression even in CRBN-knockout cells and suppressing IKZF1, IKZF3, p-GSK-3, and Myc expression. We also confirmed the efficacy of CUDC-907 in SCID mice injected with CRBN-knockout cells. It is difficult to assess how activation of NKG2D ligand by CUDC-907 contributes to tumor suppression in the xenograft model. Previous reports on NK cells during breast cancer cell growth and metastasis in SCID mice have shown that these mice have certain residual immunity, which included an NK cell-mediated response [55]. Thus, there exists a possibility that the tumor transplanted in SCID mice was attacked by residual NK cells, in addition to the direct effect of CUDC-907. Additionally, CUDC-907 was cytotoxic to primary cells from MM patients, including those resistant to various lenalidomide treatment regimens. According to the data from a pharmacokinetics study involving CUDC-907 [52], the in vitro concentration of CUDC-907 used in the current study was similar to that detected in the plasma of six patients (0–20 ng/mL, 0–39.3 nM). This concentration was observed on day 15 after receiving 60 mg of oral CUDC-907 daily for 5 days, followed by a 2-day intermittent break [52]. Furthermore, Phase I trial data indicated that the most common adverse effects of CUDC-907 were diarrhea, fatigue, nausea, and thrombocytopenia. However, these effects were relatively rare [thrombocytopenia (18%), neutropenia (7%), hyperglycemia (7%), and diarrhea (5%)] [52].

## Conclusions

**Fig. 9:**
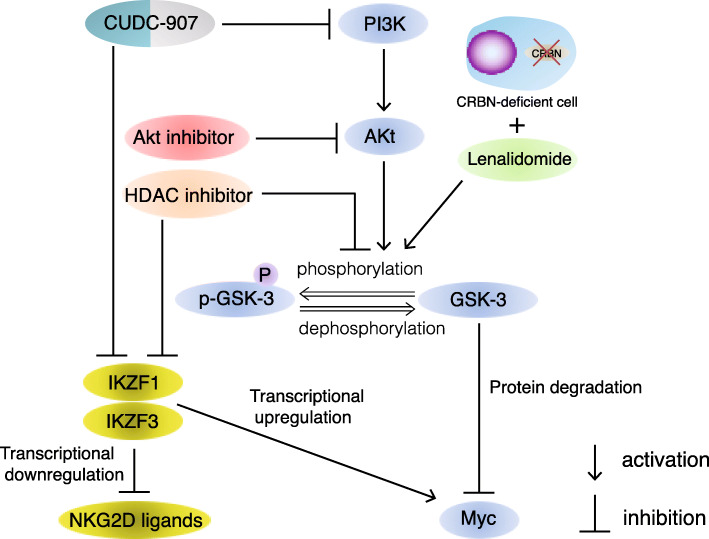
Proposed mechanism of HDAC, Akt, and PI3K inhibition in MM cells. Lenalidomide increases p-GSK-3 levels, which functions to stabilize c-Myc and enhance MM cell survival, when CRBN expression is reduced. HDAC inhibitor treatment downregulates IKZF1/3, thereby inducing c-Myc downregulation and MICA upregulation. PI3K or Akt inhibitors downregulate p-GSK-3 and c-Myc expression in MM cells. HDAC inhibition also downregulates p-GSK-3. CUDC-907 has the ability to enhance MICA and suppress c-Myc. CUDC-907 is a promising drug that has the advantages of dual HDAC and Akt inhibition

HDAC inhibitor treatment downregulated IKZF1 and IKZF3, inducing Myc downregulation and MICA upregulation. In turn, MICA upregulation led to enhanced ADCC activity of mAbs and NK cell activity. Moreover, HDAC or Akt inhibitors downregulated p-GSK-3, which functions to stabilize c-Myc. In turn, p-GSK-3 suppression caused c-Myc downregulation. Meanwhile, the dual PI3K and HDAC inhibitor, CUDC-907, enhanced MICA and suppressed Myc (Fig. 9). In summary, HDAC and Akt inhibitors, as well as CUDC-907, are promising drugs for cases of relapse/refractory MM, including lenalidomide resistance.

## Supplementary Information

**Additional file 1: Figure S1.** HDAC inhibitors upregulate NKG2D ligands. (a–d) MICA and MICB mRNA levels in RPMI8226 and U266 cells exposed to panobinostat/ACY-1215. Experiments were performed in triplicate (*n* = 3). (*p < 0.05, **p < 0.01, “ns” indicates no significant difference.) (e) The ratio of mRNA expression for MICA and MICB between cells exposed to panobinostat, romidepsin, ACY-1215 and those exposed to DMSO for 0 h and 4 h. (f) IKZF1 mRNA levels in U266 cells exposed to panobinostat. (**p < 0.01) (g-h) Prognostic value of NKG2D ligand expression based on MM patient clinical trial data.

**Additional file 2: Figure S2.** Effects of HDAC and Akt inhibitors on MM cell lines. (a) Western blot validation of the effects of ACY-1215 on Akt expression in RPMI8226. (b-d) Prognostic value of GSK-3 α/β and Myc expression based on MM patient clinical trial data. (e) Flow cytometry assay was performed with annexin V/PI staining. (f) U266, (g) H929 CRBN sh#395, (h) KMS-11, (i) KMS-11/BTZ, and (j) MM1.S were treated with DMSO, 2 μM ACY-1215 (ACY), 4 μM afuresertib (Afu), or a combination of ACY-1215 and afuresertib (comb) for 72 h. the average was calculated for each experiment performed in quintuplicate (*n* = 5). (*p < 0.05, **p < 0.01, “ns” indicates no significant difference).

**Additional file 3: Figure S3.** MTT proliferation assay in MM cell lines and peripheral blood mononuclear cells (PBMNCs) from healthy donors treated with CUDC-907. (a-h) each cell line was treated with DMSO, 10 nM CUDC-907, 25 nM CUDC-907, or 50 nM CUDC-907 for 72 h. the average was calculated for each experiment performed in quintuplicate (*n* = 5). (*p < 0.05, **p < 0.01, “ns” indicates no significant difference.) (i) PBMNCs were treated with DMSO, 2 μM ACY-1215 (ACY), 4 μM afuresertib (Afu), or a combination of ACY-1215 and afuresertib (comb) and 10 nM CUDC-907 (CUDC-907) for 72 h. the average was calculated for each experiment performed in quintuplicate (*n* = 5). (*p < 0.05, **p < 0.01, “ns” indicates no significant difference).

## Data Availability

The dataset supporting the conclusions of this article are included in the published article and its supplementary information files.

## Abbreviations

MM: Multiple myeloma
CRBN: Cereblon
IMiDs: immunomodulatory drugs
DAC: histone deacetylase
ADCC: antibody-dependent cellular cytotoxicity
NKG2D: natural killer group 2D
p-GSK-3: phosphorylation of glycogen synthase kinase-3
mAb: monoclonal antibodies
PIs: proteasome inhibitors
IKZF1: Ikaros family zinc finger 1
IKZF3: Ikaros family zinc finger 3
MICA: major histocompatibility complex class I-related chain A
MICB: major histocompatibility complex class I-related chain B
ULBp: UL16-binding proteins
DMSO: Dimethyl sulfoxide
qRT-PCR: Quantitative reverse transcription PCR
MTT: Methyl thiazolyl tetrazolium
E/T: effector/target
OS: overall survival
SD: standard deviation

## References

[1] Palumbo A, Anderson K (2011). Multiple myeloma. N Engl J Med.

[2] van de Donk NWCJ, Pawlyn C, Yong KL (2021). Multiple myeloma. Lancet.

[3] Singhal S, Mehta J, Desikan R, Ayers D, Roberson P, Eddlemon P, Munshi N, Anaissie E, Wilson C, Dhodapkar M, Zeldis J, Siegel D, Crowley J, Barlogie B (1999). Antitumor activity of thalidomide in refractory multiple myeloma. N Engl J Med.

[4] Lokhorst HM, Plesner T, Laubach JP, Nahi H, Gimsing P, Hansson M, Minnema MC, Lassen U, Krejcik J, Palumbo A, van de Donk NWCJ, Ahmadi T, Khan I, Uhlar CM, Wang J, Sasser AK, Losic N, Lisby S, Basse L, Brun N, Richardson PG (2015). Targeting CD38 with Daratumumab Monotherapy in multiple myeloma. N Engl J Med.

[5] Lonial S, Dimopoulos M, Palumbo A, White D, Grosicki S, Spicka I, Walter-Croneck A, Moreau P, Mateos MV, Magen H, Belch A, Reece D, Beksac M, Spencer A, Oakervee H, Orlowski RZ, Taniwaki M, Röllig C, Einsele H, Wu KL, Singhal A, San-Miguel J, Matsumoto M, Katz J, Bleickardt E, Poulart V, Anderson KC, Richardson P (2015). Elotuzumab therapy for relapsed or refractory multiple myeloma. N Engl J Med.

[6] Richardson PG, Barlogie B, Berenson J, Singhal S, Jagannath S, Irwin D, Rajkumar SV, Srkalovic G, Alsina M, Alexanian R, Siegel D, Orlowski RZ, Kuter D, Limentani SA, Lee S, Hideshima T, Esseltine DL, Kauffman M, Adams J, Schenkein DP, Anderson KC (2003). A phase 2 study of bortezomib in relapsed, refractory myeloma. N Engl J Med.

[7] Richardson PG, Schlossman RL, Weller E, Hideshima T, Mitsiades C, Davies F, LeBlanc R, Catley LP, Doss D, Kelly K, McKenney M, Mechlowicz J, Freeman A, Deocampo R, Rich R, Ryoo JJ, Chauhan D, Balinski K, Zeldis J, Anderson KC (2002). Immunomodulatory drug CC-5013 overcomes drug resistance and is well tolerated in patients with relapsed multiple myeloma. Blood.

[8] Schey SA, Fields P, Bartlett JB, Clarke IA, Ashan G, Knight RD, Streetly M, Dalgleish AG (2004). Phase I study of an immunomodulatory thalidomide analog, CC-4047, in relapsed or refractory multiple myeloma. J Clin Oncol.

[9] Attal M, Lauwers-Cances V, Hulin C, Leleu X, Caillot D, Escoffre M, Arnulf B, Macro M, Belhadj K, Garderet L, Roussel M, Payen C, Mathiot C, Fermand JP, Meuleman N, Rollet S, Maglio ME, Zeytoonjian AA, Weller EA, Munshi N, Anderson KC, Richardson PG, Facon T, Avet-Loiseau H, Harousseau JL, Moreau P, IFM 2009 Study (2017). Lenalidomide, Bortezomib, and dexamethasone with transplantation for myeloma. N Engl J Med.

[10] Dimopoulos MA, Oriol A, Nahi H, San-Miguel J, Bahlis NJ, Usmani SZ, Rabin N, Orlowski RZ, Komarnicki M, Suzuki K, Plesner T, Yoon SS, Ben Yehuda D, Richardson PG, Goldschmidt H, Reece D, Lisby S, Khokhar NZ, O’Rourke L, Chiu C, Qin X, Guckert M, Ahmadi T, Moreau P (2016). Daratumumab, Lenalidomide, and dexamethasone for multiple myeloma. N Engl J Med.

[11] Attal M, Lauwers-Cances V, Marit G, Caillot D, Moreau P, Facon T, Stoppa AM, Hulin C, Benboubker L, Garderet L, Decaux O, Leyvraz S, Vekemans MC, Voillat L, Michallet M, Pegourie B, Dumontet C, Roussel M, Leleu X, Mathiot C, Payen C, Avet-Loiseau H, Harousseau JL (2012). Lenalidomide maintenance after stem-cell transplantation for multiple myeloma. N Engl J Med.

[12] Ito T, Ando H, Suzuki T, Ogura T, Hotta K, Imamura Y, Yamaguchi Y, Handa H (2010). Identification of a primary target of thalidomide teratogenicity. Science.

[13] Ito T, Handa H (2016). Cereblon and its downstream substrates as molecular targets of immunomodulatory drugs. Int J Hematol.

[14] Lopez-Girona A, Mendy D, Ito T, Miller K, Gandhi AK, Kang J, Karasawa S, Carmel G, Jackson P, Abbasian M, Mahmoudi A, Cathers B, Rychak E, Gaidarova S, Chen R, Schafer PH, Handa H, Daniel TO, Evans JF, Chopra R (2012). Cereblon is a direct protein target for immunomodulatory and antiproliferative activities of lenalidomide and pomalidomide. Leukemia.

[15] Zhu YX, Braggio E, Shi CX, Bruins LA, Schmidt JE, Van Wier S (2011). Cereblon expression is required for the antimyeloma activity of lenalidomide and pomalidomide. Blood.

[16] Franssen LE, Nijhof IS, Couto S, Levin MD, Bos GMJ, Broijl A, Klein SK, Ren Y, Wang M, Koene HR, Bloem AC, Beeker A, Faber LM, van der Spek E, Raymakers R, Leguit RJ, Sonneveld P, Zweegman S, Lokhorst H, Mutis T, Thakurta A, Qian X, van de Donk NWCJ (2018). Cereblon loss and up-regulation of c-Myc are associated with lenalidomide resistance in multiple myeloma patients. Haematologica.

[17] Bjorklund CC, Lu L, Kang J, Hagner PR, Havens CG, Amatangelo M, Wang M, Ren Y, Couto S, Breider M, Ning Y, Gandhi AK, Daniel TO, Chopra R, Klippel A, Thakurta AG (2015). Rate of CRL4(CRBN) substrate Ikaros and Aiolos degradation underlies differential activity of lenalidomide and pomalidomide in multiple myeloma cells by regulation of c-Myc and IRF4. Blood Cancer J.

[18] Ziccheddu B, Biancon G, Bagnoli F, De Philippis C, Maura F, Rustad EH (2020). Integrative analysis of the genomic and transcriptomic landscape of double-refractory multiple myeloma. Blood Adv.

[19] Tachita T, Kinoshita S, Ri M, Aoki S, Asano A, Kanamori T, Yoshida T, Totani H, Ito A, Kusumoto S, Komatsu H, Yamagata K, Kubo K, Tohkin M, Fukuda S, Iida S (2020). Expression, mutation, and methylation of crbn-pathway genes at pre- and post-lenalidomide treatment in multiple myeloma. Cancer Sci.

[20] Gooding S, Ansari-Pour N, Towfic F, Ortiz Estévez M, Chamberlain PP, Tsai KT, Flynt E, Hirst M, Rozelle D, Dhiman P, Neri P, Ramasamy K, Bahlis N, Vyas P, Thakurta A. Multiple cereblon genetic changes are associated with acquired resistance to lenalidomide or pomalidomide in multiple myeloma. Blood.2021;137(2):232–7. 10.1182/blood.2020007081. 10.1182/blood.2020007081 PMC7893409 33443552

[21] Gotwals P, Cameron S, Cipolletta D, Cremasco V, Crystal A, Hewes B, Mueller B, Quaratino S, Sabatos-Peyton C, Petruzzelli L, Engelman JA, Dranoff G (2017). Prospects for combining targeted and conventional cancer therapy with immunotherapy. Nat Rev Cancer.

[22] West AC, Johnstone RW (2014). New and emerging HDAC inhibitors for cancer treatment. J Clin Invest.

[23] Bolden JE, Peart MJ, Johnstone RW (2006). Anticancer activities of histone deacetylase inhibitors. Nat Rev Drug Discov.

[24] West AC, Smyth MJ, Johnstone RW (2014). The anticancer effects of HDAC inhibitors require the immune system. Oncoimmunology.

[25] Lanier LL (2015). NKG2D receptor and its ligands in host defense. Cancer Immunol Res.

[26] Chan CJ, Smyth MJ, Martinet L (2014). Molecular mechanisms of natural killer cell activation in response to cellular stress. Cell Death Differ.

[27] Fernández-Messina L, Reyburn HT, Valés-Gómez M (2012). Human NKG2D-ligands: cell biology strategies to ensure immune recognition. Front Immunol.

[28] Kato N, Tanaka J, Sugita J, Toubai T, Miura Y, Ibata M, Syono Y, Ota S, Kondo T, Asaka M, Imamura M (2007). Regulation of the expression of MHC class I-related chain a, B (MICA, MICB) via chromatin remodeling and its impact on the susceptibility of leukemic cells to the cytotoxicity of NKG2D-expressing cells. Leukemia.

[29] Dhar P, Wu JD (2018). NKG2D and its ligands in cancer. Curr Opin Immunol.

[30] Anderson KC (2012). The 39th David a. Karnofsky lecture: bench-to-bedside translation of targeted therapies in multiple myeloma. J Clin Oncol.

[31] Younes H, Leleu X, Hatjiharissi E, Moreau AS, Hideshima T, Richardson P, Anderson KC, Ghobrial IM (2007). Targeting the phosphatidylinositol 3-kinase pathway in multiple myeloma. Clin Cancer Res.

[32] Maurer U, Preiss F, Brauns-Schubert P, Schlicher L, Charvet C (2014). GSK-3 - at the crossroads of cell death and survival. J Cell Sci.

[33] Lentzsch S, Chatterjee M, Gries M, Bommert K, Gollasch H, Dörken B, Bargou RC (2004). PI3-K/AKT/FKHR and MAPK signaling cascades are redundantly stimulated by a variety of cytokines and contribute independently to proliferation and survival of multiple myeloma cells. Leukemia.

[34] Bjorklund CC, Ma W, Wang ZQ, Davis RE, Kuhn DJ, Kornblau SM, Wang M, Shah JJ, Orlowski RZ (2011). Evidence of a role for activation of Wnt/beta-catenin signaling in the resistance of plasma cells to lenalidomide. J Biol Chem.

[35] Imai Y, Ohta E, Takeda S, Sunamura S, Ishibashi M, Tamura H (2016). Histone deacetylase inhibitor panobinostat induces calcineurin degradation in multiple myeloma. JCI Insight.

[36] Percie du Sert N, Hurst V, Ahluwalia A, Alam S, Avey MT, Baker M, Browne WJ, Clark A, Cuthill IC, Dirnagl U, Emerson M, Garner P, Holgate ST, Howells DW, Karp NA, Lazic SE, Lidster K, MacCallum CJ, Macleod M, Pearl EJ, Petersen OH, Rawle F, Reynolds P, Rooney K, Sena ES, Silberberg SD, Steckler T, Würbel H (2020). The ARRIVE guidelines 2.0: Updated guidelines for reporting animal research. PLoS Biol.

[37] Armeanu S, Bitzer M, Lauer UM, Venturelli S, Pathil A, Krusch M, Kaiser S, Jobst J, Smirnow I, Wagner A, Steinle A, Salih HR (2005). Natural killer cell-mediated lysis of hepatoma cells via specific induction of NKG2D ligands by the histone deacetylase inhibitor sodium valproate. Cancer Res.

[38] García-Guerrero E, Gogishvili T, Danhof S, Schreder M, Pallaud C, Pérez-Simón JA, Einsele H, Hudecek M (2017). Panobinostat induces CD38 upregulation and augments the antimyeloma efficacy of daratumumab. Blood.

[39] Fionda C, Abruzzese MP, Zingoni A, Cecere F, Vulpis E, Peruzzi G, Soriani A, Molfetta R, Paolini R, Ricciardi MR, Petrucci MT, Santoni A, Cippitelli M (2015). The IMiDs targets IKZF-1/3 and IRF4 as novel negative regulators of NK cell-activating ligands expression in multiple myeloma. Oncotarget.

[40] Nijhof IS, Groen RW, Lokhorst HM, van Kessel B, Bloem AC, van Velzen J (2015). Upregulation of CD38 expression on multiple myeloma cells by all-trans retinoic acid improves the efficacy of daratumumab. Leukemia.

[41] Ishibashi M, Soeda S, Sasaki M, Handa H, Imai Y, Tanaka N, Tanosaki S, Ito S, Odajima T, Sugimori H, Asayama T, Sunakawa M, Kaito Y, Kinoshita R, Kuribayashi Y, Onodera A, Moriya K, Tanaka J, Tsukune Y, Komatsu N, Inokuchi K, Tamura H (2018). Clinical impact of serum soluble SLAMF7 in multiple myeloma. Oncotarget.

[42] Zhan F, Huang Y, Colla S, Stewart JP, Hanamura I, Gupta S, Epstein J, Yaccoby S, Sawyer J, Burington B, Anaissie E, Hollmig K, Pineda-Roman M, Tricot G, van Rhee F, Walker R, Zangari M, Crowley J, Barlogie B, Shaughnessy JD (2006). The molecular classification of multiple myeloma. Blood.

[43] Mulligan G, Mitsiades C, Bryant B, Zhan F, Chng WJ, Roels S, Koenig E, Fergus A, Huang Y, Richardson P, Trepicchio WL, Broyl A, Sonneveld P, Shaughnessy JD, Leif Bergsagel P, Schenkein D, Esseltine DL, Boral A, Anderson KC (2007). Gene expression profiling and correlation with outcome in clinical trials of the proteasome inhibitor bortezomib. Blood.

[44] Richardson PG, Siegel DS, Vij R, Hofmeister CC, Baz R, Jagannath S, Chen C, Lonial S, Jakubowiak A, Bahlis N, Song K, Belch A, Raje N, Shustik C, Lentzsch S, Lacy M, Mikhael J, Matous J, Vesole D, Chen M, Zaki MH, Jacques C, Yu Z, Anderson KC (2014). Pomalidomide alone or in combination with low-dose dexamethasone in relapsed and refractory multiple myeloma: a randomized phase 2 study. Blood.

[45] Hideshima T, Cottini F, Nozawa Y, Seo HS, Ohguchi H, Samur MK, Cirstea D, Mimura N, Iwasawa Y, Richardson PG, Munshi NC, Chauhan D, Massefski W, Utsugi T, Dhe-Paganon S, Anderson KC (2017). p53-related protein kinase confers poor prognosis and represents a novel therapeutic target in multiple myeloma. Blood.

[46] Hesterberg RS, Beatty MS, Han Y, Fernandez MR, Akuffo AA, Goodheart WE, Yang C, Chang S, Colin CM, Alontaga AY, McDaniel JM, Mailloux AW, Billington JMR, Yue L, Russell S, Gillies RJ, Yun SY, Ayaz M, Lawrence NJ, Lawrence HR, Yu XZ, Fu J, Darville LN, Koomen JM, Ren X, Messina J, Jiang K, Garrett TJ, Rajadhyaksha AM, Cleveland JL, Epling-Burnette PK (2020). Cereblon harnesses Myc-dependent bioenergetics and activity of CD8+ T lymphocytes. Blood.

[47] Spencer A, Yoon SS, Harrison SJ, Morris SR, Smith DA, Brigandi RA, Gauvin J, Kumar R, Opalinska JB, Chen C (2014). The novel AKT inhibitor afuresertib shows favorable safety, pharmacokinetics, and clinical activity in multiple myeloma. Blood.

[48] Oki Y, Fanale M, Romaguera J, Fayad L, Fowler N, Copeland A, Samaniego F, Kwak LW, Neelapu S, Wang M, Feng L, Younes A (2015). Phase II study of an AKT inhibitor MK2206 in patients with relapsed or refractory lymphoma. Br J Haematol.

[49] Cohen P, Goedert M (2004). GSK3 inhibitors: development and therapeutic potential. Nat Rev Drug Discov.

[50] Li X, Su Y, Madlambayan G, Edwards H, Polin L, Kushner J, Dzinic SH, White K, Ma J, Knight T, Wang G, Wang Y, Yang J, Taub JW, Lin H, Ge Y (2019). Antileukemic activity and mechanism of action of the novel PI3K and histone deacetylase dual inhibitor CUDC-907 in acute myeloid leukemia. Haematologica.

[51] Li X, Su Y, Hege K, Madlambayan G, Edwards H, Knight T, Polin L, Kushner J, Dzinic SH, White K, Yang J, Miller R, Wang G, Zhao L, Wang Y, Lin H, Taub JW, Ge Y. The HDAC and PI3K dual inhibitor CUDC-907 synergistically enhances the antileukemic activity of venetoclax in preclinical models of acute myeloid leukemia. Haematologica. 2020:haematol.2019.233445. 10.3324/haematol.2019.233445. 10.3324/haematol.2019.233445 PMC8094102 32165486

[52] Younes A, Berdeja JG, Patel MR, Flinn I, Gerecitano JF, Neelapu SS, Kelly KR, Copeland AR, Akins A, Clancy MS, Gong L, Wang J, Ma A, Viner JL, Oki Y (2016). Safety, tolerability, and preliminary activity of CUDC-907, a first-in-class, oral, dual inhibitor of HDAC and PI3K, in patients with relapsed or refractory lymphoma or multiple myeloma: an open-label, dose-escalation, phase 1 trial. Lancet Oncol.

[53] Oki Y, Kelly KR, Flinn I, Patel MR, Gharavi R, Ma A, Parker J, Hafeez A, Tuck D, Younes A (2017). CUDC-907 in relapsed/refractory diffuse large B-cell lymphoma, including patients with MYC-alterations: results from an expanded phase I trial. Haematologica.

[54] Rajkumar SV, Harousseau JL, Durie B, Anderson KC, Dimopoulos M, Kyle R, Blade J, Richardson P, Orlowski R, Siegel D, Jagannath S, Facon T, Avet-Loiseau H, Lonial S, Palumbo A, Zonder J, Ludwig H, Vesole D, Sezer O, Munshi NC, San Miguel J, on behalf of the International Myeloma Workshop Consensus Panel 1 (2011). Consensus recommendations for the uniform reporting of clinical trials: report of the international myeloma workshop consensus panel 1. Blood.

[55] Dewan MZ, Terunuma H, Ahmed S, Ohba K, Takada M, Tanaka Y, Toi M, Yamamoto N (2005). Natural killer cells in breast cancer cell growth and metastasis in SCID mice. Biomed Pharmacother.

