# Supplementary figures and images for: Small-molecule HDAC and Akt inhibitors suppress tumor growth and enhance immunotherapy in multiple myeloma
Source: J Exp Clin Cancer Res. 2021 Mar 23;40:110. doi: 10.1186/s13046-021-01909-7 (PMC7989023; doi:10.1186/s13046-021-01909-7)

Supplemental Fig. 1

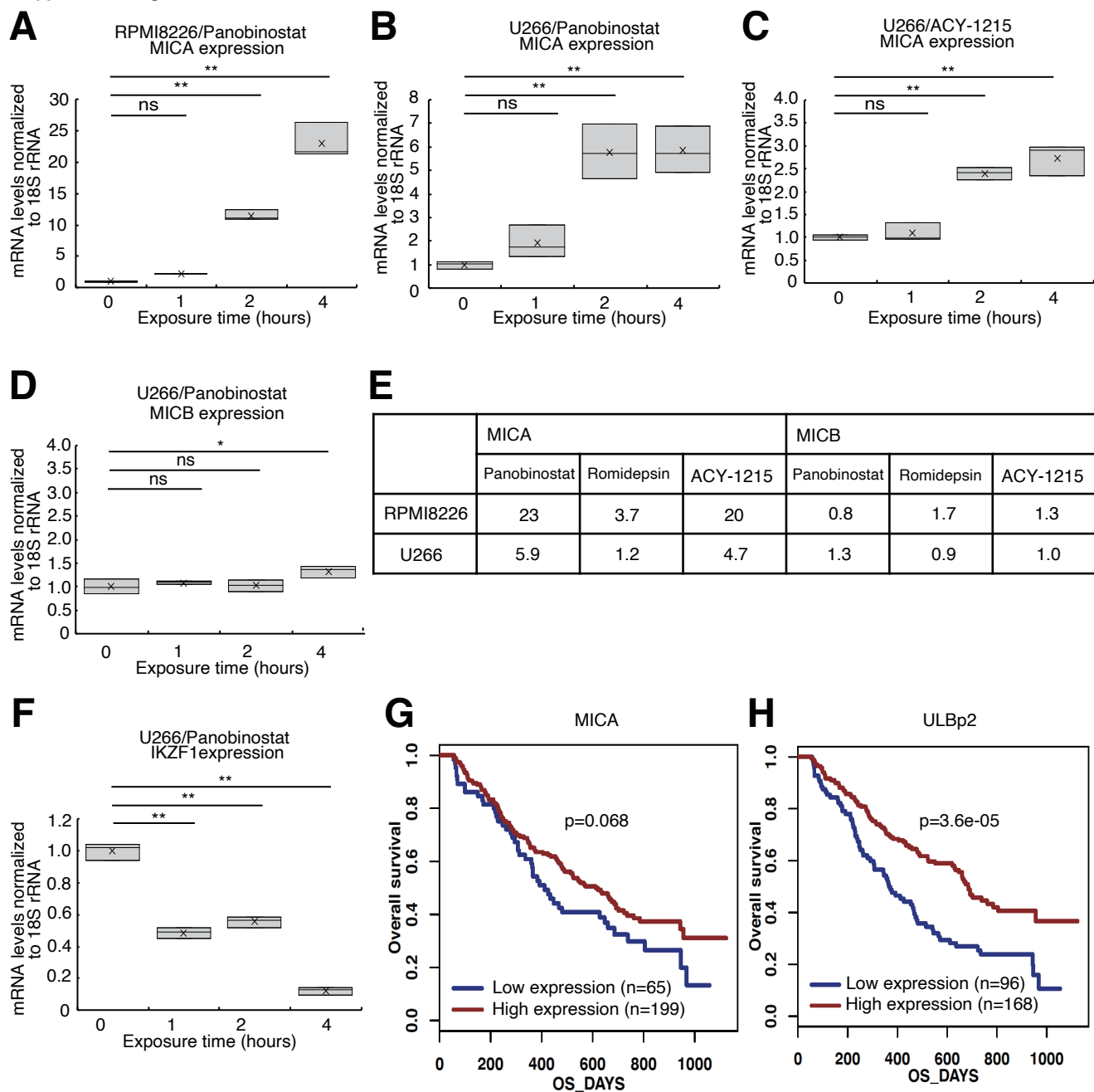

Supplement: Supplementary file 1 — Additional file 1: Figure S1. HDAC inhibitors upregulate NKG2D ligands. (a–d) MICA and MICB mRNA levels in RPMI8226 and U266 cells exposed to panobinostat/ACY-1215. Experiments were performed in triplicate (n = 3). (*p < 0.05, **p < 0.01, “ns” indicates no significant difference.) (e) The ratio of mRNA expression for MICA and MICB between cells exposed to panobinostat, romidepsin, ACY-1215 and those exposed to DMSO for 0 h and 4 h. (f) IKZF1 mRNA levels in U266 cells exposed to panobinostat. (**p < 0.01) (g-h) Prognostic value of NKG2D ligand expression based on MM patient clinical trial data. [file 13046_2021_1909_MOESM1_ESM.pdf]

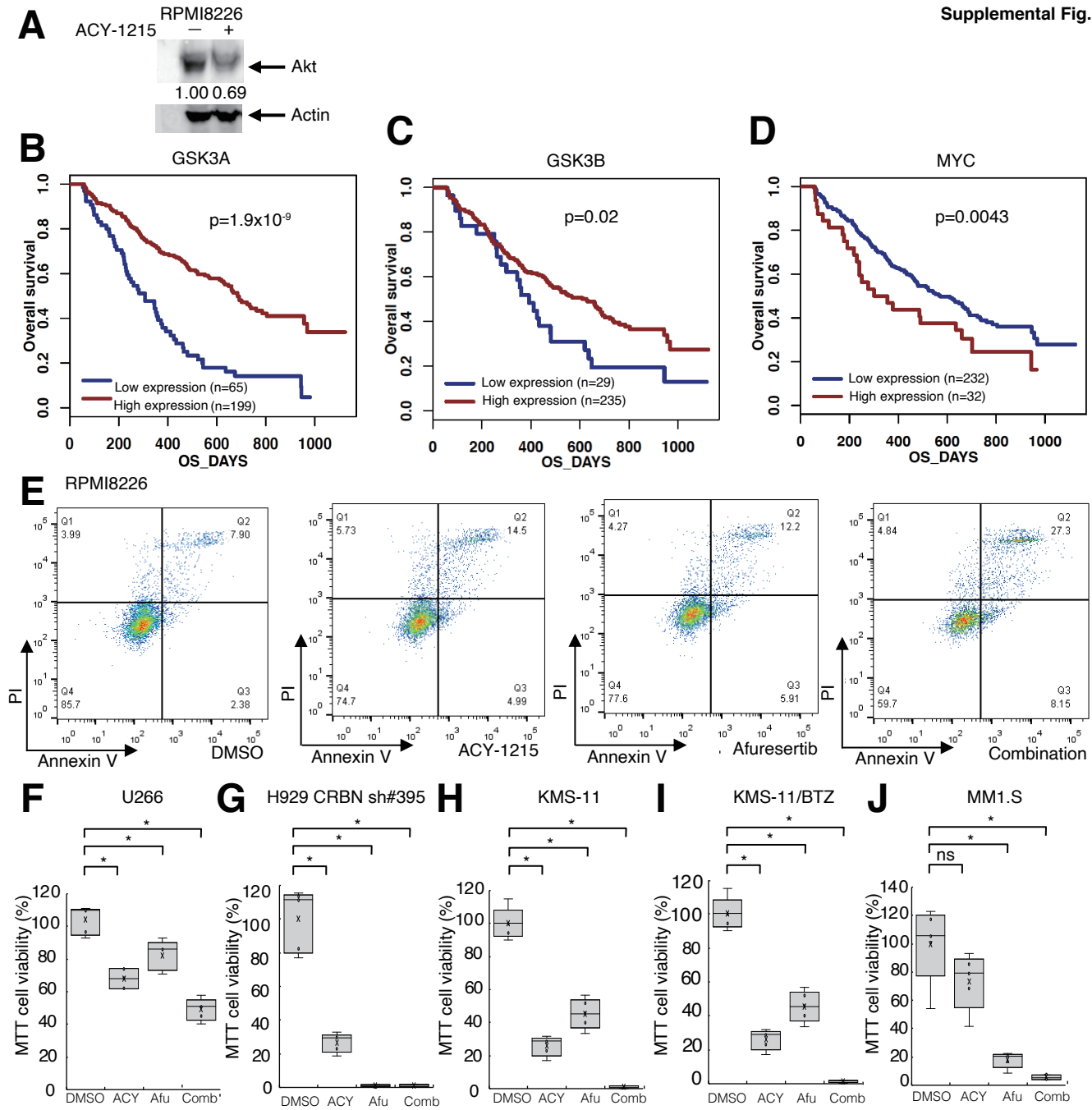

Supplement: Supplementary file 2 — Additional file 2: Figure S2. Effects of HDAC and Akt inhibitors on MM cell lines. (a) Western blot validation of the effects of ACY-1215 on Akt expression in RPMI8226. (b-d) Prognostic value of GSK-3 α/β and Myc expression based on MM patient clinical trial data. (e) Flow cytometry assay was performed with annexin V/PI staining. (f) U266, (g) H929 CRBN sh#395, (h) KMS-11, (i) KMS-11/BTZ, and (j) MM1.S were treated with DMSO, 2 μM ACY-1215 (ACY), 4 μM afuresertib (Afu), or a combination of ACY-1215 and afuresertib (comb) for 72 h. the average was calculated for each experiment performed in quintuplicate (n = 5). (*p < 0.05, **p < 0.01, “ns” indicates no significant difference). [file 13046_2021_1909_MOESM2_ESM.pdf]

Supplemental Fig. 3

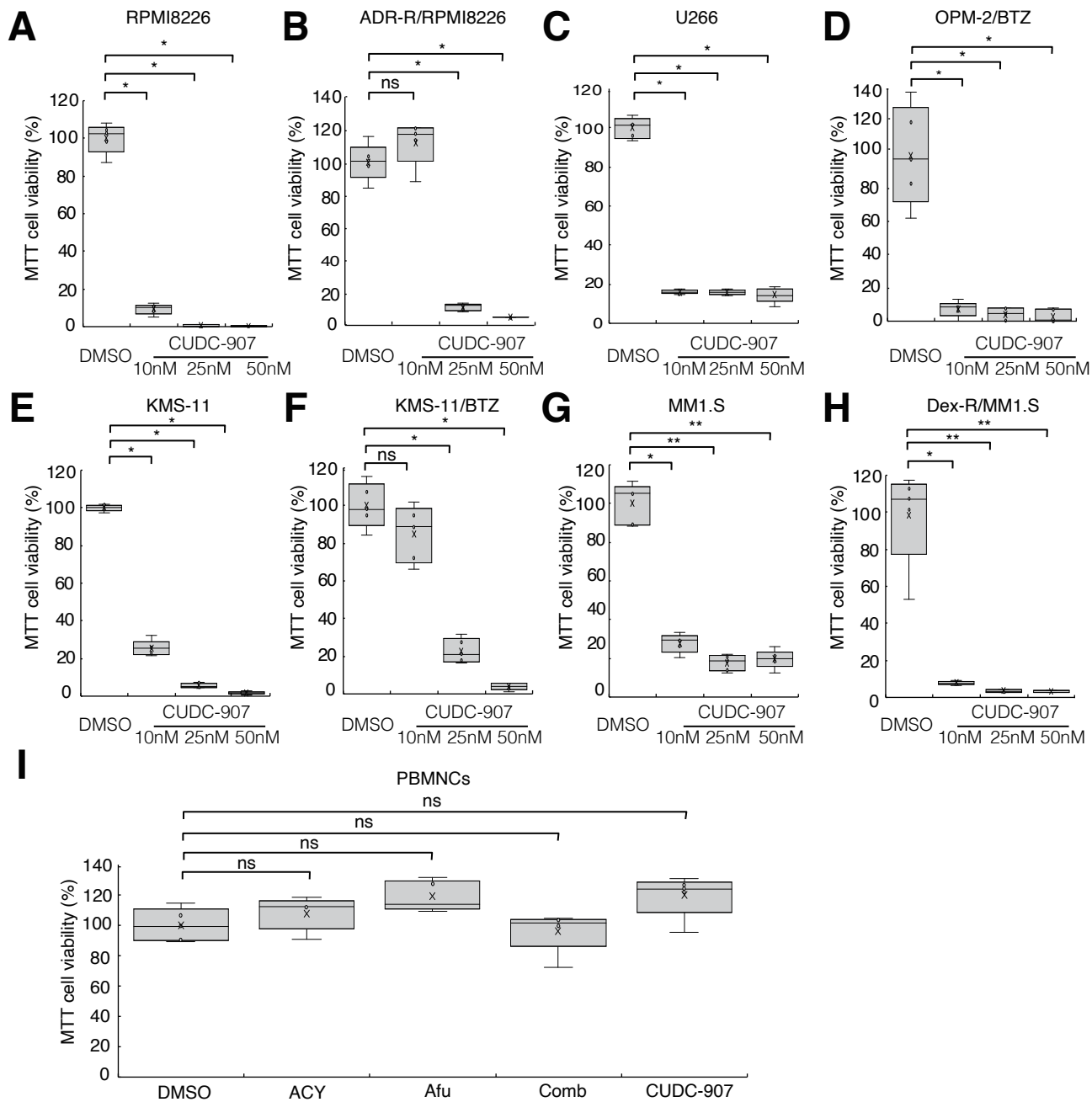

Supplement: Supplementary file 3 — Additional file 3: Figure S3. MTT proliferation assay in MM cell lines and peripheral blood mononuclear cells (PBMNCs) from healthy donors treated with CUDC-907. (a-h) each cell line was treated with DMSO, 10 nM CUDC-907, 25 nM CUDC-907, or 50 nM CUDC-907 for 72 h. the average was calculated for each experiment performed in quintuplicate (n = 5). (*p < 0.05, **p < 0.01, “ns” indicates no significant difference.) (i) PBMNCs were treated with DMSO, 2 μM ACY-1215 (ACY), 4 μM afuresertib (Afu), or a combination of ACY-1215 and afuresertib (comb) and 10 nM CUDC-907 (CUDC-907) for 72 h. the average was calculated for each experiment performed in quintuplicate (n = 5). (*p < 0.05, **p < 0.01, “ns” indicates no significant difference). [file 13046_2021_1909_MOESM3_ESM.pdf]
